# The impact of higher levels of autistic traits on risk of hikikomori (pathological social withdrawal) in young adults

**DOI:** 10.1371/journal.pone.0281833

**Published:** 2023-02-21

**Authors:** Mark Brosnan, Jeff Gavin

**Affiliations:** Department of Psychology, Centre for Applied Autism Research, University of Bath, Bath, United Kingdom; University of Catania Libraries and Documentation Centre: Universita degli Studi di Catania, ITALY

## Abstract

**Background:**

Hikikomori is an extreme state of social withdrawal, originally identified in Japan but more recently recognised internationally. Many countries imposed restrictions during the COVID-19 pandemic which may have had a detrimental impact on those at risk of hikikomori, specifically young adults and those with high levels of autistic traits.

**Aims:**

To explore whether levels of autistic traits mediate the relationship between psychological wellbeing and hikikomori risk. We also looked at whether autistic traits mediated between lockdown experiences (e.g. not leaving the house) and hikikomori risk.

**Methods:**

646 young people (aged 16–24) from a wide range of countries completed an online questionnaire assessing psychological wellbeing, autistic traits and experiences of lockdown for this cross-sectional study.

**Results:**

Autistic traits mediated the relationship between both psychological wellbeing and hikikomori risk, as well as frequency of leaving the house during lockdown and hikikomori risk. Greater hikikomori risk was associated with poor psychological wellbeing, higher autistic traits and leaving the house less frequently during the COVID-19 pandemic.

**Conclusions:**

These findings suggest similarities with Japanese hikikomori research and are consistent with suggestions that psychological wellbeing and COVID-19 restrictions are associated with increased hikikomori risk in young adults, and both associations are mediated by higher levels of autistic traits.

## Introduction

Hikikomori is a form of pathological social withdrawal or social isolation whose essential feature is physical isolation in one’s home. The person must meet the following criteria: 1. Marked social isolation in one’s home; 2. Duration of continuous social isolation for at least 6 months; 3. Significant functional impairment or distress associated with the social isolation [1–4]. In Japan, hikikomori is diagnosed in 1–2% of the general adult population [5,6], with a higher rate in younger adults specifically (4.6%, [7]). Initially, hikikomori was considered to be a ‘collectivist culture-bound’ syndrome unique to Japan. However, hikikomori is now recognised as an international phenomenon, associated with social and cultural shifts brought about by modernization, globalization, and the rise of the Internet [1,8]. Hikikomori has been identified in a wide range of countries, including some that are less collectivist (more individualist [9,10]), namely, Australia, Bangladesh, Brazil, Canada, China, France, India, Iran, Italy, Nigeria, Oman, Singapore, South Korea, Taiwan, Thailand, Ukraine, and the USA [11–13]. Hikikomori is now considered to be a boundless global, cross-cultural syndrome, most prevalent in urban areas of high-income countries [9,11,14].

Hikikomori can result in people becoming economically inactive in the long-term. Hikikomori is associated with not being in education, employment, or training (‘NEET’), which is a marker of long-term disadvantage [15]. Researchers in Japan have therefore focused upon identifying the factors that impact upon ‘hikikomori risk’ and elevated hikikomori risk has been identified in young adult males (aged 16–24; [5,14,16–18]). A study of hikikomori risk amongst Japanese university students, found that 22% of the sample were considered to be at risk of hikikomori [19].

Co-occurring mental health conditions are highly prevalent in hikikomori, however hikikomori is argued to be distinguishable from both mood disorders (such as anxiety and depression, [14]) and withdrawal conditions associated with psychotic and schizophrenia-like disorders and other personality disorders [1,12]. Specifically, hikikomori has been found to co-occur in around a third of people diagnosed with Autism Spectrum Disorder (ASD: [14,20–22]). ASD is characterized by difficulties with social communication and social interaction and restricted and repetitive patterns in behaviors, interests, and activities [23,24]. Thus, whilst there are similarities between ASD and hikikomori around social interaction, ASD is defined in terms of social difficulties whereas hikikomori is defined in terms of social withdrawal. Though ASD and hikikomori are distinguishable, the high level of co-occurrence between the conditions may be related to both groups being high in levels of autistic traits [25]. Autistic traits are argued to be distributed continuously across the population, higher in men than women, and are characterised by differences in attention to detail, attentional focus, social skills, communication, and imagination [26–29]. Autistic *traits* are not diagnostic of ASD, although autistic groups are unsurprisingly higher in autistic traits than non-autistic groups [30]. Hikikomori self-report higher levels of autistic traits than non-hikikomori in Japan [25,31].

During the recent pandemic, many countries imposed COVID-19 restrictions, some of which limited the extent to which people can leave their houses (‘lockdown’; [32,33]). This may have contributed to hikikomori risk for those susceptible to the condition [17,34–40]. Autistic traits predict increased negative emotional response to COVID-19 [41], with higher levels of autistic traits predicting a greater negative emotional response. Consequently, it has been argued that we needed to pay attention to the psychological wellbeing of those with higher levels of autistic traits during and after the COVID-19 pandemic [42,43] and to ensure that hikikomori are not ‘left behind’ as societies emerge from COVID-19 restrictions [44]. Specifically, young adults are at a critical transition stage and were disproportionately affected by COVID-19 restrictions, becoming more susceptible to both decreases in psychological wellbeing [45,46] and increases in hikikomori risk [16,34,35,47]. This study therefore sought to identify whether autistic traits mediated: 1) the relationship between wellbeing and hikikomori risk; and 2) the relationship between lockdown experiences and hikikomori risk. We hypothesised that higher levels of autistic traits would be associated with reduced psychological wellbeing relating to increased hikikomori risk. We also hypothesised that with higher level of autistic traits, there would be a greater association between impact of lockdown experiences and increased hikikomori risk.

## Materials and methods

### Participants

The inclusion/ exclusion criteria were young adults aged between 16 and 24 who identified as male or female (non-binary numbers being too small for meaningful analysis), who had access to the Internet and were fluent in written English, simplified Chinese or Hindi (see Methods). Online invitations to participate were distributed through all available social media channels. 826 participants accessed the online survey between January and April 2022, and 646 (78%) provided data for analysis.

### Measures

Participants identified their age (in years), sex (male, female), status (in education, in employment, in training, or Not in Education, Employment or Training—NEET) and their country of residence in the past year. To obtain a broad range of COVID-19 restrictions, young people were approached from a wide range of countries, including more collectivist countries typical of Asia/ Southeast Asia and more individualist countries typical of Western Europe/North America. Using the Hofstede index, each country’s rating of individualism was identified (from 0 to 100: [48]). The Hofstede index quantifies dimensions of ‘national culture’ between countries, including individualism (which is typically higher in Western countries) contrasted with collectivism (which is typically higher in Eastern countries; [20,49,50]). Authors had no access at any time to information that could identify individual participants during or after data collection.

#### Hikikomori risk

To identify hikikomori risk, we used the NEET/Hikikomori Risk (NHR) scale developed, translated and validated by Uchida and Norasakkunkit [10] with a large sample (n = 7,725). It consists of 27 items, covering three areas; 1) consciously choosing to be economically inactive (e.g., I don’t think it is necessary to find a job immediately); 2) a lack of self-competence (e.g., I feel that communicating with others is hopelessly difficult for me); and 3) unclear ambitions for the future (e.g., I don’t quite know what I want to do in the future). Participants rate how much they agree with the items on a 7-point Likert scale from “Completely disagree” to “Completely agree.” Scores could range from 1 to 7 and the scale authors report Cronbach’s α = 0.88. Uchida and Norasakkunkit [10] found that the scale effectively distinguished between those who were and were not economically inactive. Uchida and Norasakkunkit [10] also found that higher scores on this scale negatively correlated with highest educational level attained (junior school; high school; college graduate; postgraduate), positive psychological wellbeing, positive health status and number of close relationships within the community. The authors conclude that this scale is a valid measure that can distinguish those at risk of being marginalised in society and found that young adults who are NEET are disproportionally represented in the top 15% of hikikomori risk scores.

#### Psychological wellbeing

Psychological wellbeing was assessed using the SWEMWBS, which is a short version of the Warwick–Edinburgh Mental Wellbeing Scale (WEMWBS; [51,52]). Participants responded to 7 questions (e.g., I’ve been feeling optimistic about the future; I’ve been feeling useful) on a 5-point scale from ‘none of the time’ to ‘all of the time’. Participants were asked to describe their experiences over the past two weeks, and a higher score is indicative of better psychological wellbeing. The scale authors report Cronbach’s α = 0.78 to 0.80.

#### Autistic traits

Autistic traits were assessed through the AQ10 [26]. Participants responded to 10 questions (e.g., I often notice small sounds when others do not, I know how to tell if someone listening to me is getting bored) on a 4-point scale from ‘definitely agree’ to ‘definitely disagree’. Responses indicative of autistic traits were coded as 1, so overall scores could range from 0–10. Allison et al. report a Cronbach’s α = 0.45, arguing that this low value is due to the 5 subscales of the measure, and the AQ10 can be used as a single scale of autistic traits [53]. In a large-scale study of the general population (n = 44,722), Lundin et al. [54] report that the AQ-10 has adequate validity and may be used as a single measure of autistic traits.

#### Lockdown

Lockdown was defined as "the imposition of stringent restrictions on travel, social interaction, and access to public spaces." Participants were told ‘This varies between countries and may include ‘curfews’, or a variable range of restrictions (e.g., tier 1, tier 2, tier 3 etc.). Here we are asking you about any form of lockdown you may have experienced over the past year, this might include ‘total lockdown’, ‘partial lockdown’ or ‘hard’ or ‘soft’ lockdown’. Participants were asked how many months they had spent in lockdown over the past year (from: 0–12 months). This continuous variable was also dichotomised into <6months and 6+ months (see criteria for hikikomori above). Participants then identified how often they typically left their house during this time (from: 4–7 days/week; 2–3 days/week; 1 day a week or less: [1,16]).

Participants were then asked to identify CHANGES due to the impact of lockdown. A four-item scale was developed for this study: 1) How was the quality of your social life during lockdown compared to before? (e.g., fulfilment from your interaction with family/ friends—not for work/study); 2) How was the quantity of your social interaction during lockdown compared to before? (e.g., the number of times you interacted with family/ friends—not for work/study); 3) How was your mental health during lockdown, compared to before? (e.g., your mood, how you felt); 4) How was your physical health during lockdown, compared to before? (e.g., how fit you felt). Each of the four items were rated on a 7-point Likert-type scale (much less, moderately less, slightly less, the same, slightly more, moderately more, much more). Scores were averaged from all the items responded to and could range from -3 to +3. Principal components analysis confirmed the four items sat on a single factor with an Eigen value of 2.25 (56.15% of variance). The four items were taken to represent a single impact of lockdown scale, with Cronbach’s α = 0.73 for the present study.

The online survey was developed in English and then translated (and back translated) in both simplified Chinese and Hindi on the Qualtrics platform. An online invitation was distributed via social media platforms and bulletin boards in the three languages, with the appropriate link. All responses were translated into English, and the three versions were collapsed for analysis. The online survey conformed to the recommended standards for conducting and reporting web-based surveys, the Checklist for Reporting Results of Internet E-Surveys (CHERRIES: [55,56]). Ethical approval was obtained from the Psychology Research Ethics Committee of the University of Bath. All participants were adults who actively provided informed consent by checking the appropriate boxes on the online consent form, which followed a participant information page. There were no incentives for participation.

### Statistical analysis

Parametric assumptions were met for the data and mediation analyses undertaken (VIFs all around 1, suggesting no multicollinearity effects). Partial correlations (controlling for sex, age, country of residence individualism score) were conducted between the study variables and, as there were 15 comparisons, a Bonferroni correction was applied to the significance level (= .05/15 = .003). These three variables were controlled for as the literature above suggests that they may impact upon autistic traits, hikikomori and lockdown experience. Specifically, whilst young males have higher autistic traits and are at increased hikikomori risk, women are at greater risk than men of decreased psychological wellbeing during the COVID-19 pandemic [30,34,35,45,46,57]. The PROCESS macro v4.1 (model 4, 5,000 re-samples) for SPSS 26 was used for the mediation analysis [58]. Four mediation analyses were undertaken to explore whether autistic traits mediated the effect on hikikomori risk. The first analysis explored wellbeing, the second number of months in lockdown, the third days/week leaving the house and the fourth impact of lockdown. These latter three relate to the three criteria for hikikomori [1,16] to explore if autistic traits mediate the effect of lockdown on hikikomori risk. For all these analyses, the demographic variables were controlled for. Sex, age and country of residence’s individualism score were all entered as covariates.

## Results

There were 283 (43.8%) men and 363 (56.2%) women. Participants were young adults aged 16–24 (mean 20.08, sd = 1.80; which did not differ by sex t(644) = .59, p = .56). Participants were residing in one of 45 countries, largely the UK, Singapore and India (see S1 Appendix for full list). These country’s individualism ratings had a mean of 58 (sd = 29, range = 14–91). Participants self-reported that they were either: In education (526, 81%); In employment (115, 18%); In training (20, 3%); Not in Education, Employment or Training (NEET, 28, 4%) or Other (e.g., military/ national service, 24, 4%). 10% were in both education and employment. Table 1 highlights the means, standard deviations and ranges of the study variables.

**Table 1:** Distributions of the study variables.

Variable	Mean (sd)	Range
Hikikomori risk	3.35 (0.84)	1.41–6.67
Autistic traits	3.73 (1.86)	0–10
Wellbeing	2.05 (0.70)	0.14–4.00
Months_lockdown	5.84 (3.24)	0–12
Leave_house_lockdown	2.28 (0.78)	01–3
Impact_lockdown	3.17 (1.25)	01–7

Partial correlations, controlling for sex, age and country of residence individualism score were conducted between the study variables. Table 2 highlights that greater hikikomori risk was associated with higher levels of autistic traits and lower levels of psychological wellbeing. Higher levels of autistic traits were also associated with lower levels of psychological wellbeing. In terms of lockdown experiences, months in lockdown had no significant relationships and the impact of lockdown only correlated with the psychological wellbeing measure. Leaving the house less frequently, however, was associated with greater hikikomori risk and lower psychological wellbeing.

**Table 2:** Correlations between the study variables.

Variable	2.	3.	4.	5.	6.
1.Hikikomori risk	.38***	-.53***	.04	.17***	-.09
2.Autistic traits		-.32***	.02	.1	-.06
3.Wellbeing			-.05	-.12***	.31***
4.Months_lockdown				-.01	-.09
5.Leave_house_lockdown					.05
6.Impact_lockdown					

***p < .003.

**Fig 1:**
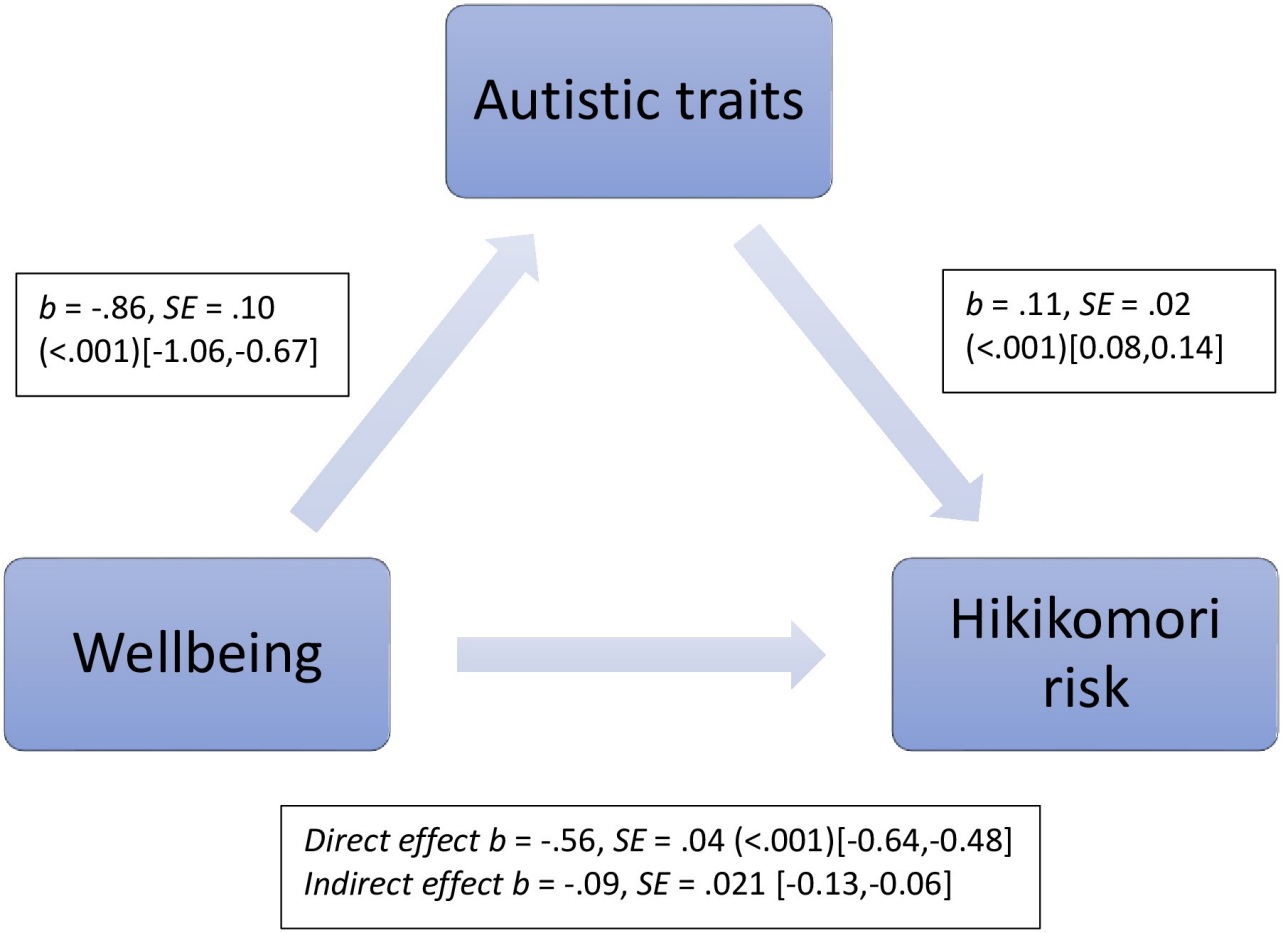
The indirect effect of autistic traits on the association between wellbeing and hikikomori risk. Regression coefficients are unstandardized. p values are shown in parentheses where applicable. 95% percentile bootstrap confidence intervals are shown in square brackets [5,000 re-samples].

The first mediation analysis identified that autistic traits significantly mediated the relationship between psychological wellbeing and hikikomori risk (sex, age and country of residence’s individualism score were covariates). Fig 1 shows both the significant direct effect between wellbeing and hikikomori risk as well as the significant indirect effect, mediated by autistic traits. Autistic traits accounted for 14% of the total combined effect (direct and indirect).

**Fig 2:**
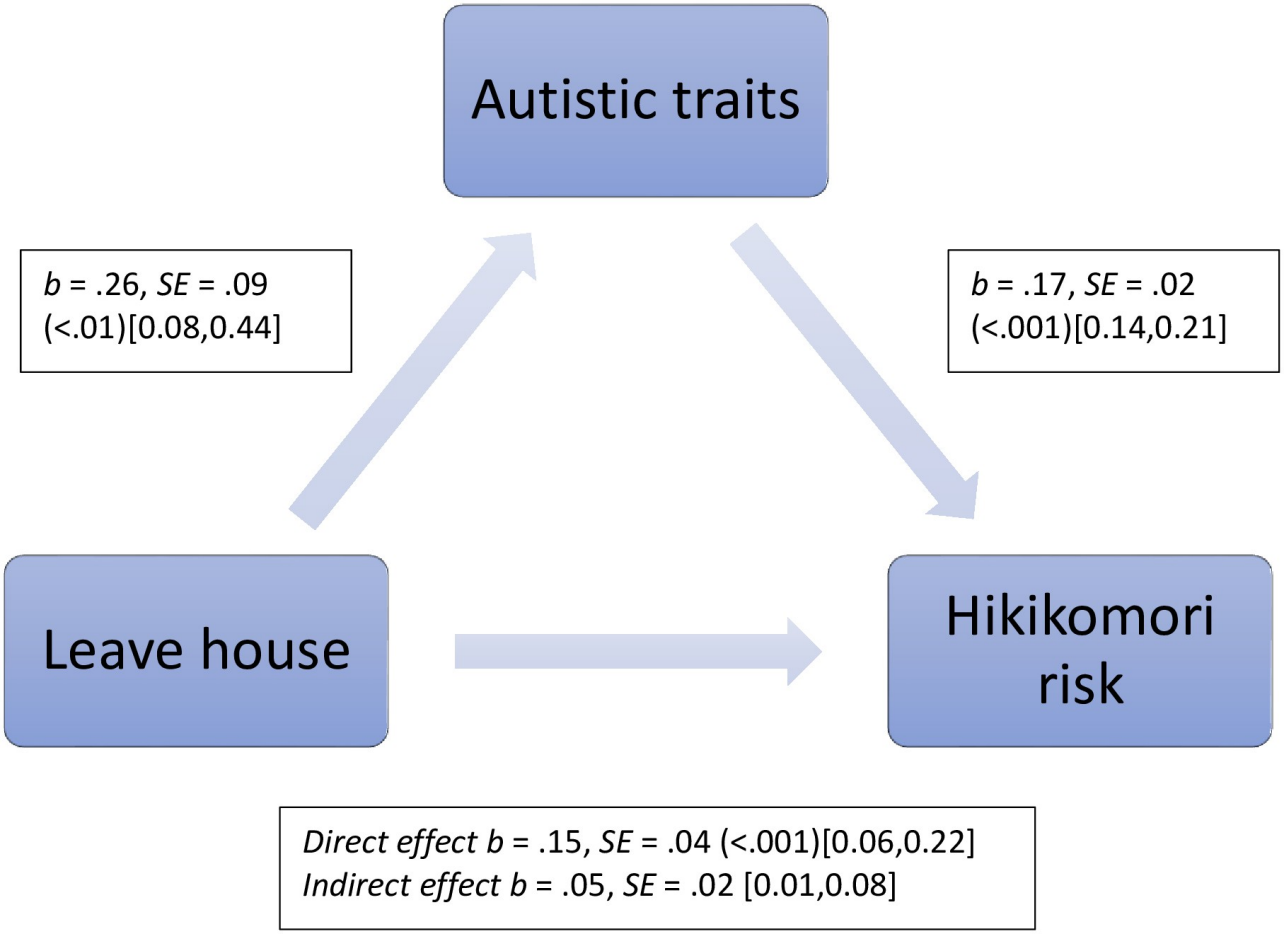
The indirect effect of autistic traits on the association between leaving the house and hikikomori risk. Regression coefficients are unstandardized. p values are shown in parentheses where applicable. 95% percentile bootstrap confidence intervals are shown in square brackets [5,000 re-samples].

Autistic traits also mediated the relationship between leaving the house during lockdown and hikikomori risk (again with sex, age and country of residence’s individualism score as covariates). Fig 2 shows both the significant direct effect between leaving the house and hikikomori risk as well as the significant indirect effect, mediated by autistic traits. Autistic traits accounted for 23% of the total combined effect (direct and indirect). There were no significant direct or indirect (mediated by autistic traits) effects between months in lockdown and hikikomori risk or impact of lockdown and hikikomori risk (all p>.05; except the relationship between autistic traits and hikikomori risk, as above).

A MANCOVA (controlling for sex, age, country of residence individualism score) was conducted to explore whether hikikomori risk, autistic traits, wellbeing, leaving the house and impact of lockdown differed by the dichotomised variable of months in lockdown (< 6 months (%) and 6+ months (%)–as this is the proposed cut off for hikikomori criteria). All the analyses were non-significant (p>.05) except for impact of lockdown (F(1,622) = 9.86, p = .002). Those in lockdown for 6+ months experienced a greater negative impact of lockdown. Replacing the continuous months in lockdown variable with the dichotomised months in lockdown variable did not affect the mediation analysis reported above.

Finally, in the present study, 57% of those self-identifying as NEET were in the top 15% of hikikomori scores, which is a significantly greater proportion than non-NEET young adults in the top 15% of hikikomori risk scores (Chi^2^(1) = 31.16, p < .001).

## Discussion

Hikikomori is a state of pathological social withdrawal associated with poor psychological wellbeing and the present study investigated the mediating effects of autistic traits on this relationship. Experiences of lockdown restrictions during the COVID-19 pandemic may also impact upon hikikomori risk, and the present study also investigated whether autistic traits mediated this relationship. The results demonstrated both a significant direct effect of psychological wellbeing on hikikomori risk as well as a significant indirect effect mediated by autistic traits. Similarly, there was a significant direct relationship between leaving the house less and hikikomori risk as well as a significant indirect effect, mediated by autistic traits. The number of months spent in lockdown and the impact of lockdown did not have a significant association with hikikomori risk (direct or indirect).

These findings are consistent with research from Japan that has identified associations between hikikomori risk and psychological wellbeing as well as those with higher autistic traits having higher risk of hikikomori [14,25,31,59]. The present study had an international sample, and the findings are consistent with those from Japan which support the contention that hikikomori is an international phenomenon rather than ‘culture bound’ [1,20]. The individualism score (of the countries resided in) in the present study was a covariate in the analysis, which is again consistent with the findings being independent of collectivist/individualist cultural differences.

Concern has also been raised that lockdown restrictions during COVID-19 may have exacerbated the risk of hikikomori, particularly in young adults [5,14,16,18]. Using the three criteria for hikikomori proposed by Kato et al. [1,16], the present study found specifically that lower levels of leaving the house was associated with increased hikikomori risk (consistent with Kato et al. [1,2]). Clearly, causality needs to be established, but increasing the frequency of leaving the house could be a focus for future research exploring amelioration of high levels of hikikomori risk, post-COVID-19 restrictions. Reduced frequency of leaving the house for more than 6 months is a criterion for hikikomori [1,16], suggesting that support to leave the house for young people at risk of hikikomori should be provided at the earliest opportunity. Developing a better understanding of the relationship between higher levels of autistic traits and reduced frequency of leaving the house can inform the provision of support. Future research can explore which aspects of autistic traits specifically relate to frequency of leaving the house and hikikomori risk. For example, in addition to social and communication skills, the AQ also assesses attention to detail, attentional focus, and imagination [26,27]. Autistic traits correlate with technological capabilities [60] and technology-based solutions, such as Pokémon Go, have been proposed to encourage hikikomori out of the house [61,62].

As noted, however, causality needs to be established, as a reduced level of leaving the house may be an index of other factors, for example a marker of social isolation. A recent intervention for hikikomori in Japan has been to send people into the houses of hikikomori [63]. Gavin and Brosnan [47] also found that using social media ameliorated hikikomori risk during lockdown. This could indicate that leaving the house less frequently was an indicator of reduced social interaction generally that increased hikikomori risk. In the present study, however, the measure of impact of lockdown asked explicitly about the quality and quantity of social interaction, and this was not associated with hikikomori risk. Importantly, the measure of the impact of lockdown significantly correlated with the established measure of psychological wellbeing. The negative impact of lockdown was also greater for those who had experienced 6+ months of lockdown, which is consistent with the criteria for hikikomori proposed by Kato et al. [1,16]. However, in the present study, the impact of lockdown measure and months in lockdown (continuous or dichotomised as a variable) were not associated with hikikomori risk.

Taken together, the results suggest that psychological wellbeing generally (rather than related to the impact of lockdown specifically) and a reduced level of leaving the house are two key indicators of hikikomori risk, and both are mediated by autistic traits. This is consistent with the proposal that COVID-19 restrictions detrimentally impact upon those susceptible to hikikomori [16,34,35], and that we need to pay attention the psychological wellbeing of those with higher levels of autistic traits and to ensure that hikikomori are not ‘left behind’ as societies emerge from COVID-19 restrictions [42–44]. The importance of this is reflected in the significantly higher proportion of those at risk of hikikomori who self-reported being NEET (consistent with [10]), which can be a marker of long-term disadvantage [15]. In addition, autistic individuals typically have higher levels of autistic traits than non-autistic groups [30] and previous research has suggested that autism may be associated with hikikomori [12,20–22]. The present study, however, suggests greater hikikomori risk is mediated by higher levels of autistic traits and future research can tease out the relative contribution of autistic traits and autism diagnostic status on hikikomori (see [60] for a similar approach).

Systematic review of research exploring the impact of the COVID-19 pandemic on the mental health and wellbeing of the general population has identified high heterogeneity from very detrimental to somewhat beneficial, and this heterogeneity cannot be attributed to observed population or country characteristics [64]. The present study controlled for country characteristics (individualism) and suggests that individual characteristics (such as levels of autistic traits) can explain some of the heterogeneity in the impact of COVID-19 pandemic on mental health and wellbeing. The present study also recruited young adults, and this age range has been found to be at higher risk of increased depressive symptoms due to the COVID-19 pandemic [36–39]. Other individual factors such as job security and social economic status were also related to increased depressive symptoms during the COVID-19 pandemic [39], which is consistent with the direct effect between wellbeing and hikikomori in the present study and participants not in employment, education or training (NEET) being at higher hikikomori risk. Gavin and Brosnan [47] suggest that online social interaction may be a useful strategy for reducing hikikomori in NEETs, however individual factors around internet addiction may also be relevant for future research [65,66].

The means reported in Table 1 are comparable to those reported by the authors of the measures [1,10,16,26,51,52] indicating that the present sample were within the expected ranges of the measures under investigation. There were also several limitations to the present study that limit the generalisability of the findings. The measures in the present study were general (e.g., months in lockdown over the past year…) which is a limitation of the study, as is the short versions of the assessments (AQ10; SWEMWEBS) used and the cross-sectional design. Using longer versions of the autistic traits measures can inform subscale analysis in future research to identify which aspects of autistic traits mediate relationships with hikikomori risk. The questionnaires were also translated for the present study which represents a further limitation. The ‘impact of lockdown’ variable was developed for this study, which is another limitation, although it did significantly correlate with a well-validated measure of psychological wellbeing. An online convenience sampling methodology was used which enabled access to an international sample. However, a limitation is that there was no way of determining response rate (other than comparison to those who started but did not complete the online survey). This is a significant limitation to understanding both the generalisability and representativeness of the sample, who were mostly in education. We did not perform an apriori sample size estimation and specific diagnoses were not exclusion criteria, which are additional limitations of this exploratory study. Anxiety and depression related disorders specifically have been associated with increased symptom severity during the COVID-19 pandemic [40]. Additionally, countries vary on more dimensions than the single index of individualism used in the present study [64], and these limitations need to be borne in mind. Care should also be taken comparing these findings to those of Japan where formal diagnostic criteria have been developed for hikikomori [1,16]. The present study indexed self-reports of these criteria, rather than a more clinical approach of identifying how severe the impact of hikikomori may be on the individual.

As an exploratory study, however, the findings do highlight potentially interesting parallels with research that has been conducted over a longer time period in Japan. The significance of autistic traits, psychological wellbeing and frequency of leaving the house when considering hikikomori risk are relevant internationally as societies emerge from post-COVID-19 restrictions.

## Conclusions

This international sample has many similarities with findings from Japanese studies, supporting the contention that hikikomori is an international phenomenon. The relationship between wellbeing and hikikomori risk was mediated by autistic traits. Individuals with higher levels of autistic traits who do not leave the house were at higher risk of hikikomori.

## Supporting information

S1 Appendix (DOCX)
