# Supplementary material for: The impact of higher levels of autistic traits on risk of hikikomori (pathological social withdrawal) in young adults
Source: PLoS One. 2023 Feb 21;18(2):e0281833. doi: 10.1371/journal.pone.0281833 (PMC9942989; doi:10.1371/journal.pone.0281833)
Supplement: S1 Appendix — (DOCX) [file pone.0281833.s001.docx]

**Appendix A: Country and individualism score**

Austria 55; Bahrain 25; Belgium 75; Bulgaria 30; Brunei 25; Canada 80; China 20; Czech 58; Dubai 25; Egypt 25; Finland 63; France 71; Germany 67; Greece 35; Hong Kong 25; India 48; Indonesia 14; Ireland 70; Italy 76; Jamaica 39; Japan 46; Kenya 25; Malaysia 26; Monaco 71; Netherlands 80; Poland 60; Portugal 27; Peru 16; Philippines 32; Qatar 25; Romania 30; Russia 39; Saudi Arabia 25; Singapore 20; Slovenia 27; South Africa 65; South Korea 18; Spain 51; Switzerland 68; Sri Lanka 35; Thailand 20; Turkey 37; UAE 25; UK 89; USA 91.
